# Poikilocytosis in Rabbits: Prevalence, Type, and Association with Disease

**DOI:** 10.1371/journal.pone.0112455

**Published:** 2014-11-17

**Authors:** Mary M. Christopher, Michelle G. Hawkins, Andrew G. Burton

**Affiliations:** 1 Department of Pathology, Immunology and Microbiology, University of California Davis, Davis, CA, 95616, United States of America; 2 Department of Medicine and Epidemiology, University of California Davis, Davis, CA, 95616, United States of America; 3 William R. Pritchard Veterinary Medical Teaching Hospital, University of California Davis, Davis, CA, 95616, United States of America; GI Lab, United States of America

## Abstract

Rabbits (*Oryctolagus cuniculus*) are a popular companion animal, food animal, and animal model of human disease. Abnormal red cell shapes (poikilocytes) have been observed in rabbits, but their significance is unknown. The objective of this study was to investigate the prevalence and type of poikilocytosis in pet rabbits and its association with physiologic factors, clinical disease, and laboratory abnormalities. We retrospectively analyzed blood smears from 482 rabbits presented to the University of California-Davis Veterinary Medical Teaching Hospital from 1990 to 2010. Number and type of poikilocytes per 2000 red blood cells (RBCs) were counted and expressed as a percentage. Acanthocytes (>3% of RBCs) were found in 150/482 (31%) rabbits and echinocytes (>3% of RBCs) were found in 127/482 (27%) of rabbits, both healthy and diseased. Thirty-three of 482 (7%) rabbits had >30% acanthocytes and echinocytes combined. Mild to moderate (>0.5% of RBCs) fragmented red cells (schistocytes, microcytes, keratocytes, spherocytes) were found in 25/403 (6%) diseased and 0/79 (0%) healthy rabbits (P = 0.0240). Fragmentation and acanthocytosis were more severe in rabbits with inflammatory disease and malignant neoplasia compared with healthy rabbits (P<0.01). The % fragmented cells correlated with % polychromasia, RDW, and heterophil, monocyte, globulins, and fibrinogen concentrations (P<0.05). Echinocytosis was significantly associated with renal failure, azotemia, and acid-base/electrolyte abnormalities (P<0.05). Serum cholesterol concentration correlated significantly with % acanthocytes (P<0.0001), % echinocytes (P = 0.0069), and % fragmented cells (P = 0.0109), but correlations were weak (Spearman ρ <0.02). These findings provide important insights into underlying pathophysiologic mechanisms that appear to affect the prevalence and type of naturally-occurring poikilocytosis in rabbits. Our findings support the need to carefully document poikilocytes in research investigations and in clinical diagnosis and to determine their diagnostic and prognostic value.

## Introduction

Poikilocytosis is the presence of abnormally shaped erythrocytes in peripheral blood. Identification of poikilocytes is an important part of blood smear evaluation because shape changes often are associated with specific diseases, providing clues to underlying pathogenesis and facilitating diagnosis and treatment. Poikilocytes can result from biochemical changes, toxins, or physical damage to erythrocytes; regardless of cause, they can shorten erythrocyte survival and contribute to anemia [1], [2]. In healthy pigs and young goats and calves, poikilocytes (acanthocytes, echninocytes, dacryocytes) are found normally in peripheral blood, without apparent pathologic consequence [1].

Rabbits are popular companion animals, are raised for meat, and are used extensively as animal models of human disease, including atherosclerosis, disorders of lipid metabolism, diabetes, and cardiovascular disease [3]–[5]. Acanthocytes have been observed in blood smears from healthy laboratory rabbits [6]. Described in 1967 as “thorn apple”-shaped red blood cells (RBCs), numerous acanthocytes (or acantho-echinocytes) were observed together with small microcytes about one-fourth the size of a normal red cell [7]. Sanderson and Phillips [8] later described echinocytes, acanthocytes, and schistocytes in cardiac and arterial blood smears from healthy New Zealand White rabbits; they concluded that the poikilocytes were probably artifact and “indicative of a poorly prepared smear”. However, while echinocytes can be the result of artifact (crenation), acanthocytes and schistocytes are pathologic cells that involve splenic remodeling and occur with in vivo fragmentation or membrane lipid abnormalities [1], [9], [10]. Acanthocytes, echinocytes, and occasionally schistocytes have been associated with liver disease and hypercholesterolemia in humans [2], [10], [11], dogs [12], and cats [13] and with disseminated intravascular coagulation and some types of neoplasia in dogs [12], [14], [15]. Red cell shape abnormalities (mainly acanthocytes) also are well described in laboratory rabbits [16], [17] and dogs [18] fed atherogenic diets. In our hematology laboratory we have occasionally observed poikilocytes in companion rabbits presented to the University of California-Davis Veterinary Medical Teaching Hospital (VMTH). To the authors’ knowledge, no studies have been done to quantify poikilocytes in healthy rabbits or to investigate possible links between poikilocytes and clinical or biochemical variables. A better understanding of red cell morphology in rabbits would be beneficial to researchers and clinicians alike.

The objectives of this study were to retrospectively characterize the prevalence, type, and severity of poikilocytes in a large cohort of rabbits and to investigate associations between poikilocytes and physiologic factors (age, sex, and breed), clinical disease, and CBC and biochemical findings. We hypothesized that poikilocytes in rabbits would be associated with specific diseases or laboratory abnormalities.

## Materials and Methods

### Study design and data collection

Electronic medical records from the University of California-Davis VMTH were searched retrospectively for rabbit visits between 1990 and 2010 at which a complete blood count (CBC) was done. Cases were excluded if a blood smear was not available or if a clinical diagnosis was not provided. Repeat visits by the same rabbit were excluded. Patient number, date, signalment (age, sex, breed), clinical and pathologic diagnoses, and CBC and biochemistry results were recorded. Age was reported in years and categorized as adult (≥1 year old) or juvenile (<1 year). Clinical diagnoses were those reported in the visit summary by the clinician, and were based on the results of physical examination, laboratory tests, imaging, and occasionally, histopathology. Pathology results (biopsy and necropsy) were recorded if they were obtained within 6 months of the CBC and/or were relevant to the primary clinical disease. Healthy rabbits were those presented for routine physical examination or elective spay or neuter.

### Hematology and biochemistry data

From January, 1990 to September, 2001 hematology results were obtained using a Baker Systems 9110 Plus hematology analyzer (BioChem ImmunoSystems Inc., Allentown, PA, USA). From September, 2001 to December, 2010 hematology results were obtained using an ADVIA 120 hematology system (Siemens Healthcare Diagnostics Inc., Tarrytown, NY, USA) with the rabbit setting in MultiSpecies System Software. Differential counts were obtained manually by counting 200 leukocytes in Wright-Giemsa-stained blood smears. Total plasma protein concentration was determined by refractometry and fibrinogen concentration was determined using the heat precipitation method. Hemolysis (pink plasma color) was qualitatively evaluated as mild, moderate, or marked. Biochemical results were obtained on a Roche Hitachi 917 analyzer (Roche Diagnostics Corporation, Indianapolis, IN, USA) from 2006 to 2010, Hitachi 717^c^ from 1997 to 2005, and Coulter Dacos analyzer (Coulter Electronics Inc, Hialeah, FL, USA) from 1990 to 1996. When instruments were upgraded, results were calibrated to retain consistency in results between analyzers.

### Quantitation of poikilocytes

Original blood samples had been collected and processed according to our laboratory’s standard operating procedure. Whole blood was placed into tubes containing EDTA and smears were prepared, air-dried, and stained by certified medical technologists within ∼1 hour of collection. Poikilocytes were reported semiquantiatively (eg, few, moderate, many) by the technologists. Smears were coverslipped prior to storage in the laboratory slide archive.

On each original stained smear, 2000 RBCs were counted and characterized at 1000X magnification by a senior clinical pathology resident (AGB) blinded to information about the rabbit. Poikilocytes were defined based on standard morphology and counting was limited to representative monolayer fields in which about half the erythrocytes were touching but did not overlap [1], [19], [20]. In severely anemic patients, counting was limited to areas where erythrocytes were separated by no more than one cell diameter. The number and type of poikilocytes were recorded and expressed as a percentage of RBCs. Poikilocytosis was subsequently classified as none (0%), rare (0.05–0.5%), mild (>0.5–3%), moderate (>3–10%), or marked (>10%). The number and percentage of polychromatophils were also recorded.

A board-certified clinical pathologist (MMC) independently determined % poikilocytes in a subset of the smears, also in a blinded manner; results from the two observers were averaged for analysis. The subset included a random sample consisting of every 10^th^ slide (according to laboratory accession number) and all samples where the qualitative poikilocyte results in the original hematology report were widely discrepant from the quantitative results.

### Statistical methods

Data were compiled in an Excel (Microsoft Corp, Redmond, WA, USA) spreadsheet and examined for aberrant entries. Statistical analyses were done using JMP 10.0.0 (SAS Institute Inc, Cary, NC, USA). Poikilocyte percentages were tested for normality by examination of histograms and Shapiro-Wilk tests. Differences in % poikilocytes between groups were evaluated using Wilcoxon/Kruskal-Wallis rank sums tests. Rabbits with none, rare, and mild (few) poikilocytes were combined and compared with those having moderate to marked poikilocytosis. Differences in age and hematologic and biochemical data were analyzed using Student’s *t* test or ANOVA. Chi square analysis was used to compare nominal data. A multivariate model using Spearman’s rank test was used to evaluate correlations among % poikilocytes and CBC and biochemistry variables. Principal component analysis was done using % poikilocytes and selected CBC and biochemistry variables. A P-value of.05 was used to indicate statistical significance.

## Results

Nine-hundred-seventy-five rabbit visits with at least partial CBC results were identified during the 20-year period. Of these, 406 samples were excluded as repeat CBCs; 48 were excluded because smears were missing; 25 were excluded because they were native brush rabbits (*Sylvalagus bachmani*); and 14 were excluded because a clinical diagnosis was not reported. In total, 482 rabbits were included in the study.

Rabbits ranged from 3 months to 12 years of age (median 4 years), with 428 adults and 54 juveniles. Rabbits included 205 females (113 intact, 92 spayed) and 277 males (137 intact, 140 castrated). Rabbit breeds included Netherland Dwarf (n = 64), Lop (60), Mini-Lop (30), Mini-Rex (28), Holland Lop (20), New Zealand White (16), Dutch (13), Rex (10), Angora (8), French Lop (8), Flemish Giant (6), English Spot (5), English Lop (4), Dwarf (4), Dwarf Hotot (3), Lionhead (2), Chinchilla Rabbit (2), 1 each Dwarf Lionhead, Finnish Giant, Florida White, Havana, Hottot, Jersey Wooly, Lop-eared Angora, Norwegian Dwarf, and crossbred (21); breed was not specified for 170 rabbits.

Of the 482 rabbits, 79 were healthy and 403 were diseased. The mean (± SD) age of healthy rabbits (1.6±1.9 years) was significantly lower than that of diseased rabbits (4.6±2.8 years) (P<0.0001, Student’s t test). No difference was found in the proportion of females and males or in breed distribution between healthy and diseased rabbits.

### Prevalence and type of poikilocytes

A total of 155/482 (32%) smears were quantified by two observers, with good agreement in % poikilocytes (average difference 0.05%±5% over a range of 0 to 70%). In the remaining smears, quantitative findings were similar to poikilocytes noted (or not) in the original laboratory report.

**Figure 1:**
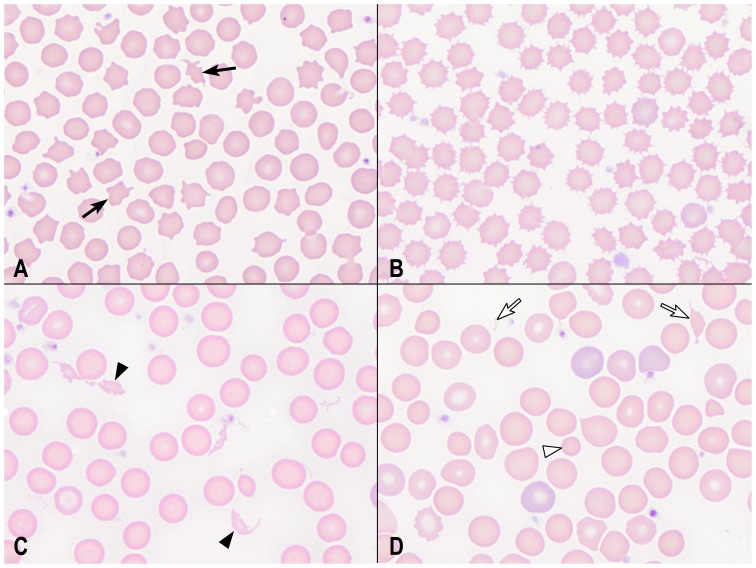
Poikilocytes in blood smears from rabbits. (A) Acanthocytes (arrows) in a healthy rabbit; (B) echinocytes in a rabbit with renal failure; (C) schistocytes (closed arrowheads) in a rabbit with a dental abscess; and (D) spherocytes (open arrowhead) and schistocytes (open arrows) in a rabbit with a mandibular abscess. Wright-Giemsa stain. Scale bar = 10 µm.

**Figure 2:**
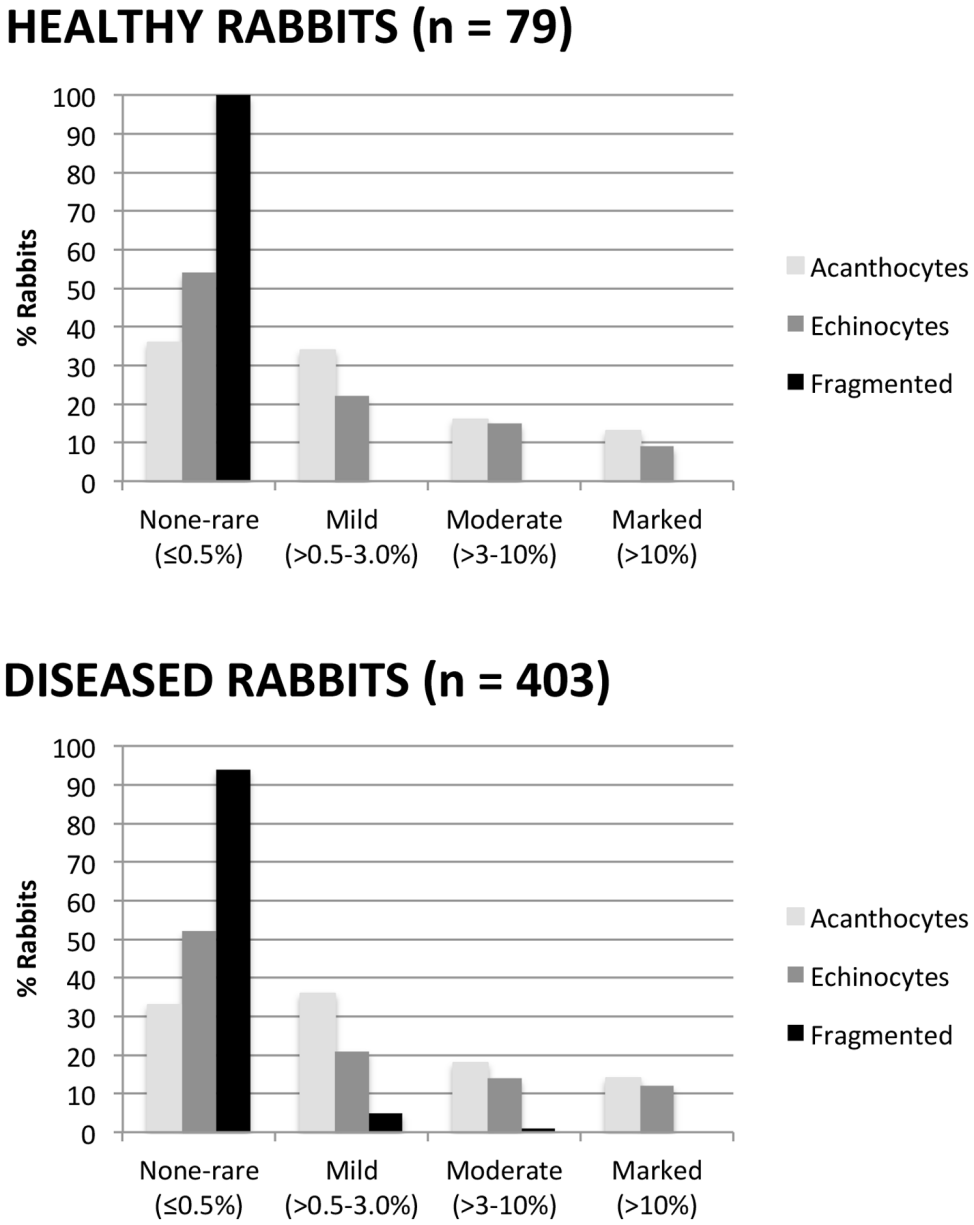
Prevalence of poikilocytes in 492 rabbits. Fragmented cells include schistocytes, keratocytes, microcytes, and spherocytes.

**Figure 3:**
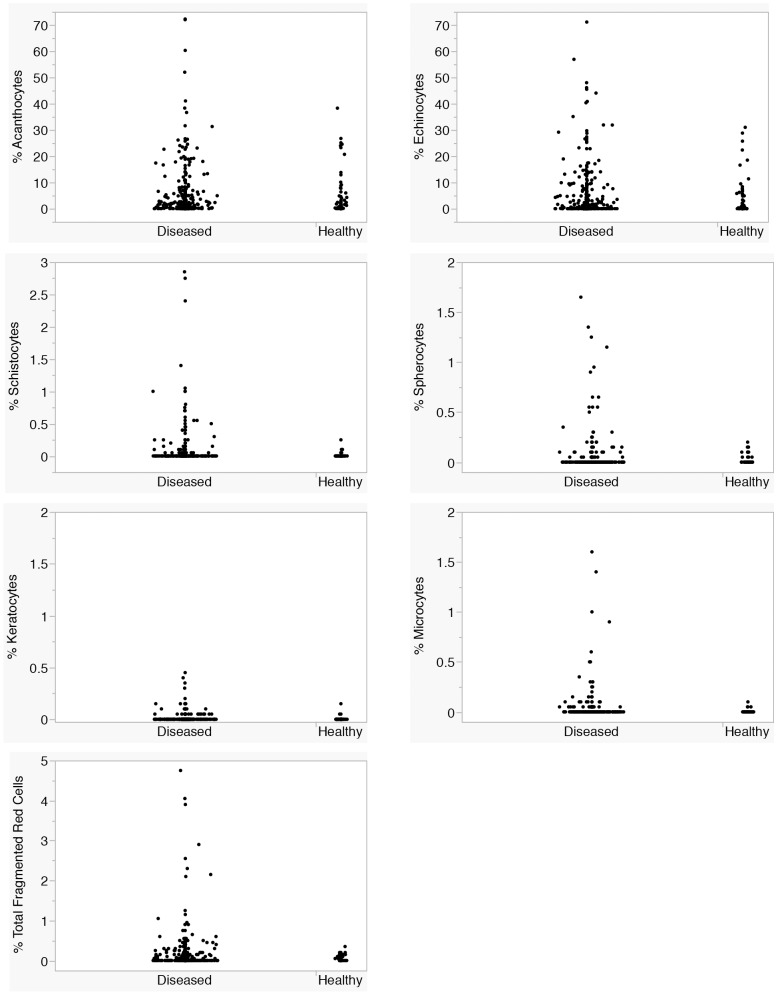
Dot plots of % poikilocytes in samples from healthy (n = 79) and diseased (n = 403) rabbits. Significant differences between healthy and diseased rabbits were observed for % schistocytes, % keratocytes, % microcytes, and % total fragmented cells (P<.05, Wilcoxon).

A majority of rabbits 251/482 (52%) had none, rare, or mild (<3%) poikilocytosis; 90/482 (19%) rabbits had moderate and 141 (29%) had marked poikilocytosis. Acanthocytes and echinocytes were the most frequently observed poikilocytes, with no significant difference between healthy and diseased rabbits (Figures 1–3). One-hundred-fifty of 482 (31%) rabbits had moderate to marked acanthocytosis and 127/482 (27%) had moderate to marked echinocytosis. Of these, 10 (2%) rabbits (including 1 healthy rabbit) had >30% acanthocytes and 11 (2%) rabbits (including 2 healthy rabbits) had >30% echinocytes. Acanthocytes and echinocytes often were observed together (Spearman ρ = 0.3896, P<0.0001) and overlapped in morphology; 33/482 (7%) rabbits had >30% acanthocytes and echinocytes combined. Acanthocyte morphology ranged from cells with one to two elongated blebs to multiple, smooth to sharply spiculated and irregularly-placed projections. Echinocytes had fine- to blunt-tipped, evenly spaced, short projections; those with blunt-tipped projections sometimes occurred together with irregularly-spiculated acanthocytes. No significant difference in the percentage of acanthocytes and echinocytes was observed between healthy and ill rabbits.

Schistocytes, microcytes, keratocytes, and spherocytes (subsequently combined as “fragmented” red cells) were observed in low numbers, with 457/482 (95%) rabbits having none to rare fragmented cells and 25/482 (5%) having mild to moderate fragmentation (Figures 1–3). Only diseased rabbits had mild to moderate fragmentation (P = 0.0024, Chi square), and a higher percentage of fragmented cells was found in diseased compared with healthy rabbits (P = 0.0240, Wilcoxon) (Figure 2). Fragmented red cells usually were found together with acanthocytes, with or without echinocytes. Percent fragmented cells correlated significantly with % acanthocytes (Spearman’s ρ = 0.4400, P<0.0001) and to a lesser extent with % echinocytes (Spearman’s ρ = 0.2861, P<0.0001. Microcytes often were very small, less than one-fourth the diameter of a normal red cell. Ovalocytes, dacryocytes, blister cells, and knizocytes were observed in low numbers in a few rabbits, as were occasional stomatocytes, target cells and ghost cells; because of their low frequency, these poikilocytes were not analyzed further.

No significant difference in % poikilocytes was observed between samples with none (n = 374), slight (n = 101), or moderate (n = 7) sample hemolysis. Further, neither moderate to marked acanthocytosis or echinocytosis nor mild to moderate fragmentation were associated with the presence of sample hemolysis. None of the 7 samples with moderate hemolysis had increased fragmentation and only 3/98 samples with mild to moderate fragmentation had slight hemolysis.

### Poikilocytes and physiologic factors

Associations between poikilocytes and physiologic factors were evaluated in clinically healthy rabbits (n = 79). Healthy rabbits included 47 adults and 32 juveniles ranging from 3 months to 8 years of age. No significant difference in % poikilocytes or in the proportion of samples with moderate to marked poikilocytosis was found between adult and juvenile rabbits; no correlation was found between age and % poikilocytes.

Healthy rabbits included 32 females (28 intact, 4 spayed) and 47 males (35 intact, 12 castrated). Female rabbits had a slightly higher % echinocytes (median 0.8%, range 0–31.0%) than male rabbits (median 0.1%, range 0–28.8%) (P = 0.0309, Wilcoxon). A higher proportion of females than males had moderate to marked acanthocytosis (41/32, 44% vs 9/47, 15%) and echinocytosis (12/32, 84% vs 7/47, 15%) (P<0.05, Chi square).

Healthy rabbits included Netherland Dwarf (14), Mini-Rex (5), Dutch (4), Holland Lop (4), and Lop (4), New Zealand White (2), Mini-Lop (2), Lionhead (2), English Spot (2), 1 each of Angora, Dwarf, Dwarf Lionhead, English Lop, Flemish Giant, Hottot, and Rex; and cross-breed (5), breed was not reported for 28 rabbits. No significant breed difference was found in % poikilocytes or in the proportion of samples with moderate to marked poikilocytes, however samples sizes were small.

### Poikilocytes and disease

Diseased rabbits were classified into 12 organ groups based on primary diagnosis (Table 1). Pathology results were available for 101/403 (25%) diseased rabbits, including 70 necropsy (with histopathology), 27 biopsy, and 4 cytology results. Rabbits also were grouped based on the specific disease process; groups with 10 or more rabbits were analyzed statistically for associations with poikilocytosis (Table 2).

**Table 1:** Primary diagnosis by organ system for the 482 rabbits in the study (1990 to 2010).

Organ System	Disease	Clinical Diagnosis	Pathology Diagnosis	Total
Bone and joint	Fracture	14	0	14
	Degenerative joint disease	5	2	7
	Luxation	2	0	2
	Neoplasia (sarcoma, squamous cell carcinoma)	0	2	2
	Other (lameness, multifocal bony lesions)	2	0	2
Cardiovascular	Myxomatous valve degeneration	2	1	3
	Cardiomyopathy	1	1	2
	Arrythmia	1	0	1
Dental	Malocclusion/periodontitis	37	1	38
	Mandibular abscessation/osteomyelitis	29	9	38
Gastrointestinal	GI stasis	25	0	25
	Enteritis/gastritis/typhylitis/colitis	2	9	11
	Diarrhea	5	0	5
	Trichobezoar	0	3	3
	Abscess	1	1	2
	Neoplasia (papilloma, adenocarcinoma)	0	2	2
	Other (dysbiosis, cecal impaction)	4	0	4
Hemolymphatic	Mediastinal thymoma	2	2	4
	Lymphoma	0	3	3
	Regenerative anemia	1	0	1
Hepatic	Enzymopathy	4	0	4
	Hepatitis/cholangiohepatitis	0	3	3
	Torsion with necrosis	0	2	2
	Neoplasia (cystadenocarcinoma)	0	1	1
Neurologic	Encephalitozoonosis	0	6	6
	Ataxia/paresis/paralysis	6	0	6
	Myelopathy	5	0	5
	Vestibular disease	3	0	3
	CNS disease	3	0	3
	Neoplasia (pineocytoma)	0	1	1
	Bacterial meningoencephalitis	0	1	1
	Brain hemorrhage	0	1	1
	Other (lumbosacral disease, seizures, head trauma)	3	0	3
Ophthalmic	Keratitis/uveitis/ulcer	8	0	8
	Glaucoma/cataracts	5	0	5
	Chemosis/conjunctivitis	5	0	5
	Dacryocystitis	5	0	5
	Iris granuloma	3	0	3
	Other (corneal fibrosis, laceration, dystrichia, epicorneal membrane)	4	0	4
Reproductive	Uterine disease (endometritis, cysts, hydrometra, mass, varices)	4	1	5
	Neoplasia (testicular granular cell tumor)	0	1	1
Respiratory	Upper respiratory tract disease	20	3	23
	Bronchopneumonia	3	5	8
	Lung abscessation+pneumonia	0	4	4
	Neoplasia (carcinoma, granular cell tumor)	0	2	2
	Other (nasal mass, lung consolidation, stridor)	3	0	3
Skin/subcutis	Otitis	13	1	14
	Cellulitis (bite wounds, myiasis)	7	3	10
	Dermatitis, nonparasitic	9	5	14
	Dermatitis, parasitic	10	0	10
	Pododermatitis	6	0	6
	Abscess, soft tissue	3	3	6
	Laceration	6	0	6
	Neoplasia, malignant (sarcoma, squamous cell carcinoma)	0	7	7
	Neoplasia, benign (lipoma, polyp)	1	2	3
	Other (myositis/fibrosis, cutaneous mass)	3	0	3
Urinary	Renal failure	7	7	14
	Urolithiasis	8	0	8
	Urine sludge	7	0	7
	Cystitis	4	0	4
Other	Anorexia, weight loss	5	0	5
	Abdominal mass	1	0	1
	Hypercalcemia	1	0	1

**Table 2:** The proportion of rabbits with specific diseases having moderate to marked acanthocytosis or echinocytosis and mild to moderate fragmentation as compared with healthy rabbits.

Disease†	Clinical Diagnosis	Pathologic Diagnosis	AcanthocytosisMod-Mkd	EchinocytosisMod-Mkd	FragmentationMild-Mod
Abscess	32	18	26/50 (52%)**	14/50 (28%)	7/50 (14%)***
Bronchopneumonia	3	9†	8/12 (67%)*	3/12 (25%)	1/12 (8%)**
Cellulitis§	7	3	7/10 (70%)**	4/10 (40%)	2/10 (20%)***
Dental (non-abscess)	37	1	11/38 (29%)	14/38 (36%)	2/38 (5%)*
Dermatitis	19	5	5/24 (21%)	6/24 (25%)	1/24 (4%)
Fracture	12	2	3/14 (21%)	2/14 (14%)	0/14 (0%)
GI inflammation§	2	9	4/11 (36%)	2/11 (18%)	1/11 (9%)**
GI stasis	25	0	5/25 (20%)	6/25 (24%)	0/25 (0%)
Neoplasia, malignant	0	22	5/22 (22%)	3/22 (14%)	2/22 (9%)**
Otitis	13	1	3/14 (21%)	3/14 (21%)	0/14 (0%)
Renal failure	7	7¶	4/14 (28%)	8/14 (57%)*	1/14 (7%)*
URTD	20	3	7/23 (30%)	7/23 (30%)	0/23 (0%)
Healthy	79	0	23/79 (29%)	19/79 (24%)	0/79 (0%)

*P<.05; **P<.01; ***P<.001.

† Four rabbits with bronchopneumonia also had lung abscessation; 4 had confirmed sepsis.

§ Eight of 10 cases of cellulitis were septic (myiasis or bacterial); GI inflammation was septic in 7/11 cases (5 with intralesional bacteria, 1 with Coccidia, 1 with Coccidia and Giardia.

¶ Two rabbits with renal failure also had pneumonia.

GI indicates gastrointestinal; URTD indicates upper respiratory tract disease.

No difference in the % or severity of acanthocytes and echinocytes was found on the basis of organ system, but a significantly lower % fragmented cells was found in healthy rabbits and in rabbits with ophthalmic disease compared with other organ systems (P<.05, Wilcoxon). Moderate to marked acanthocytosis and mild to moderate fragmentation were observed significantly more often in rabbits with septic and inflammatory disorders compared with healthy rabbits (Table 2). A significantly higher % of echinocytes (P = 0.0086) and moderate to marked echinocytosis (Table 2) were observed in rabbits with renal failure compared with healthy rabbits.

### Poikilocytes and laboratory abnormalities

Significant differences were observed in laboratory values in rabbits with moderate to marked acanthocytosis or echinocytosis and with mild to moderate fragmentation (Table 3). Because few reticulocyte counts (n = 18) and band heterophils (n = 35) were reported, these analytes were not analyzed statistically.

**Table 3:** Hematologic and biochemical values (mean ± SEM) in 482 rabbits based on severity of poikilocytosis.

Analyte	Acanthocytosis			Echinocytosis			Fragmentation		
	Moderate-marked	None-mild	P value*	Moderate-marked	None-mild	P value*	Mild-moderate	None-rare	P value*
RBC (X10^6^/µl)	5.8±0.1	5.8±0.1	–	6.0±0.1	5.7±0.1	0.0055	5.3±0.2	5.8±0.1	0.0241
HCT (%)	36.7±0.5	37.2±0.3	–	38.5±0.5	36.6±0.3	0.0012	34.3±1.1	37.2±0.2	0.0124
HGB (g/dl)	12.3±0.1	12.5±0.1	–	12.9±0.2	12.3±0.1	0.0055	11.1±0.4	12.5±0.1	0.0006
MCV (fl)	63.7±0.3	64.6±0.2	0.0454	64.6±0.3	64.2±0.2	–	65.3±0.8	64.3±0.2	–
MCH (pg)	21.4±0.1	21.7±0.1	–	21.5±0.1	21.6±0.1	–	20.9±0.3	21.6±0.1	0.0243
MCHC (g/dl)	33.6±0.1	33.6±0.1	–	33.4±0.1	33.7±0.1	–	32.1±0.3	33.7±0.1	<0.0001
RDW (%)	13.8±0.1	13.2±0.1	0.0008	13.4±0.1	13.4±0.1	–	16.1±0.3	13.3±0.1	<0.0001
POLY (%)	1.3±0.1	1.2±0.04	–	1.3±0.1	1.2±0.04	–	1.8±0.1	1.2±0.03	0.0002
WBC (/µl)	7677±264	7509±178	–	8036±287	7393±171	–	8493±646	7510±151	–
HET (/µl)	3780±211	3663±142	–	4022±230	3585±137	–	4914±515	3632±120	0.0159
LYM (/µl)	2940±143	2970±96	–	3008±156	2944±93	–	2562±351	2983±82	–
MONO (/µl)	525±38	510±26	–	606±41	483±24	0.0116	679±94	506±22	–
EOS (/µl)	89±8	84±5	–	80±8	88±5	–	81±19	86±4	–
BASO (/µl)	314±19	261±13	0.0226	278±21	277±12	–	238±47	280±11	–
PLT (X10^3^/µl)	630±25	579±16	–	609±27	588±15	–	764±62	586±13	0.0057
MPV (fl)	7.4±0.2	7.2±0.1	–	7.7±0.2	7.1±0.1	0.0105	8.4±0.5	7.2±0.1	0.0228
TPP (g/dl)	7.2±0.1	7.1±0.04	–	7.3±0.1	7.1±0.1	–	7.1±0.1	7.2±0.04	–
FIB (mg/dl)	303±16	312±10	–	340±17	298±10	0.0377	408±38	304±9	0.0076
AG (mmol/L)	25.4±0.6	24.8±0.4	–	27.1±0.6	24.1±0.4	0.0001	26.3±1.7	25.0±0.3	–
Na (mmol/L)	144.0±0.4	143.6±0.2	–	144.6±0.4	143.4±0.2	0.0079	143.2±1.0	143.8±0.2	–
K (mmol/L)	4.6±0.1	4.4±0.1	0.0202	4.6±0.1	4.4±0.1	0.0271	5.0±0.2	4.4±0.04	0.0077
Cl (mmol/L)	102.9±0.4	102.5±0.3	–	103.6±0.5	102.3±0.3	0.0246	102.8±1.2	102.6±0.2	–
HCO3 (mmol/L)	20.3±0.5	20.9±0.3	–	18.5±0.4	21.6±0.3	<0.0001	19.1±1.2	20.8±0.2	–
CA (mg/dl)	14.7±0. 1	14.4±0.1	–	14.5±0.1	14.5±0.1	–	14.2±0.3	14.5±0.1	–
PHOS (mg/dl)	3.6±0.2	3.7±0.1	–	4.0±0.2*	3.5±0.1	0.0331	3.5±0.5	3.7±0.1	–
BUN (mg/dl)	24.3±2.0	23.2±1.3	–	26.7±2.1	22.4±1.3	–	29.1±5.0	23.3±1.1	–
CREA (mg/dl)	1.3±0.1	1.4±0.1	–	1.7±0.1	1.3±0.1	0.0148	1.6±0.3	1.4±0.1	–
GLU (mg/dl)	164.7±4.0	166.4±2.7	–	158.3±4.3	168.6±2.6	0.0424	146.2±10.2	166.8±2.3	0.0482
T. PROT (g/dl)	7.3±0.2	6.9±0.1	–	7.0±0.2	7.1±0.1	–	6.9±0.5	7.1±0.1	–
ALB (g/dl)	5.3±0.1	5.2±0.1	–	5.1±0.1	5.2±0.1	–	4.4±0.2	5.3±0.04	<0.0001
GLOB (g/dl)	1.7±0.1	1.97±0.1	–	1.9±0.1	1.7±0.1	–	2.5±0.2	1.7±0.05	0.0010
CHOL (mg/dl)	58.1±3.9	43.5±2.6	0.0044	57.3±4.2	44.3±2.6	0.0094	93.5±9.9	45.6±2.2	<0.0001
T. BILI (mg/dl)	0.1±0.1	0.2±0.1	–	0.1±0.1	0.2±0.1	–	0.1±0.2	0.2±0.1	–
ALT (U/L)	50±17	81±11	–	57±18	77±11	–	66±44	71±10	–
AST (U/L)	44±62	129±42	–	64±67	116±41	–	63±159	104±36	–
ALP (U/L)	62±8	77±6	–	60±9	77±6	–	87±22	71±5	–
CK (U/L)	1244±430	2198±298	–	2429±465	1680±289	–	6567±1110	1658±246	<0.0001
GGT (U/L)	10±2	12±1	–	11±2	11±1	–	19±5	11±1	–
N	118–150	245–332	–	101–127	262–355	–	16–25	346–457	–

*Student’s t test between rabbits having moderate-marked (>3% of RBCs) vs none-mild (≤3% of RBCs) acanthocytes or echinocytes, or mild-moderate (>0.5% of RBCs) vs none-rare (≤0.5% of RBCs) fragmented red cells.

– indicates no significant difference.

Based on Spearman rank correlation for all rabbits combined, % acanthocytes correlated positively with % polychromasia, red cell distribution width (RDW), and mean platelet volume (MPV) (P<0.02); % echinocytes correlated positively with RBC count, hematocrit (HCT), hemoglobin concentration (HGB), mean cell hemoglobin concentration (MCHC), MPV, anion gap, and sodium, potassium, chloride, and creatinine concentrations, and negatively with bicarbonate and glucose concentrations (P<0.01); % fragmented cells correlated positively with % polychromasia, MCHC, RDW, and heterophil, monocyte, and potassium concentrations, and negatively with bicarbonate and albumin concentrations (<0.02). Cholesterol correlated significantly with % acanthocytes (P<0.0001), % echinocytes (P = 0.0069), and % fragmented cells (P = 0.0109), but was not highly predictive (Spearman ρ <0.2).

**Figure 4:**
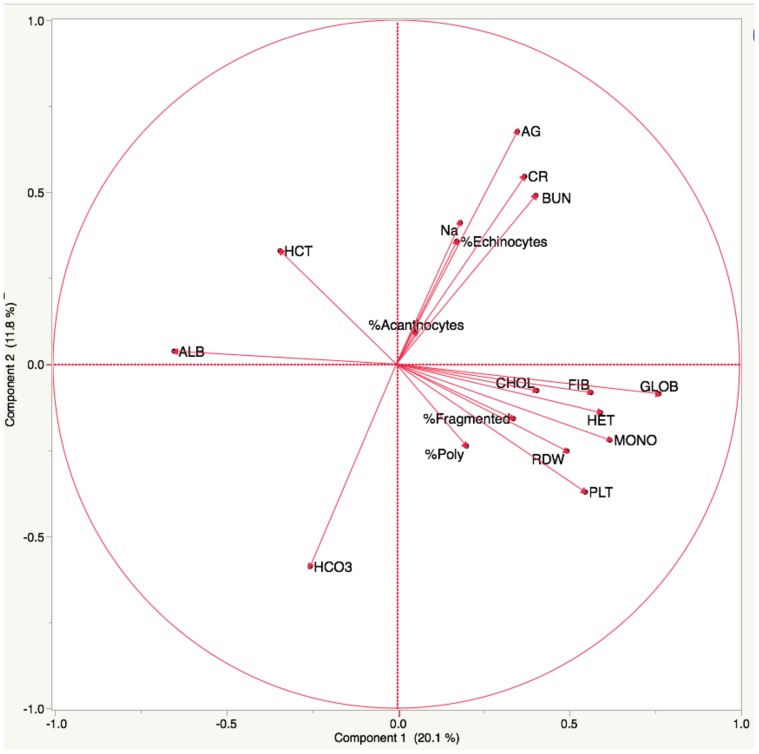
Principal component analysis of % poikilocytes and selected laboratory values. Two primary components were identified, in the right upper (component 2) and right lower (component 1) quadrants. The longer the arrow, the stronger the correlation. Analytes in left quadrants are negatively correlated with those in the diagonal quadrant.

We used principal component analysis to further visualize correlation patterns (Figure 4). In component 1, fragmented cells, and to a lesser extent acanthocytes, correlated positively with polychromasia, heterophils, monocytes, fibrinogen, globulins, and cholesterol; and negatively with albumin and HCT. In component 2, echinocytes correlated positively with azotemia, sodium, potassium, and anion gap; and negatively with bicarbonate.

## Discussion

To our knowledge, this study is the first to quantify red cell morphology in a large population of rabbits. Acanthocytes and echinocytes comprised >3% of RBCs in about one-third of healthy and diseased rabbits and were slightly more frequent in healthy female than male rabbits. Fragmented red cells (schistocytes, microcytes, keratocytes, spherocytes) occurred in low numbers and were more frequently observed in diseased rabbits, especially those with abscesses, bronchopneumonia, cellulitits, gastrointestinal inflammation, and malignant neoplasia. In addition, moderate to marked echinocytosis was associated with renal failure and electrolyte abnormalities. These findings suggest common pathophysiologic mechanisms of poikilocyte formation in sepsis (fragmentation) and uremia (echinocytes). Our findings warrant further investigation into the pathogenesis of red cell shape change in rabbits and assessment of the diagnostic or prognostic value of poikilocytes.

Acanthocytes and echinocytes were the most frequent poikilocytes observed in rabbits in this study; they often occurred together and formed a morphologic spectrum that sometimes made differentiation difficult [12]. Acanthocytes have irregularly spaced, blunt-tipped projections, an irreversible shape change that usually results from altered membrane cholesterol or phospholipids [1], [10]–[12]. Echinocytes have evenly spaced, narrow-tipped, reversible projections that form in the presence of fatty acids, lysophospholipids, and a variety of chemical agents [1], [9], [11]. Although acanthocytes (and sometimes echinocytes) are a pathologic finding, echinocytes (crenation) can be an artifact of excess EDTA, prolonged blood storage, or slow drying of smears [1]. Acanthocytes (or acantho-echinocytes) were first reported in healthy laboratory rabbits nearly 50 years ago [7] and have been observed anecdotally in companion rabbits. However, because of the frequent attribution of echinocytes to artifact [8], [9] and because, based on our results, only a small proportion of rabbits had many acanthocytes and/or echinocytes, these poikilocytes may be overlooked or considered as insignificant in the routine examination of rabbit blood. Our findings emphasize the importance of documenting all poikilocytes, including echinocytes.

Rabbits fed high cholesterol (atherogenic) diets routinely develop acanthocytes (“spur cells”) or echinocytes [16], [17], [21]–[23]. Serum cholesterol concentrations in the rabbits in this study also were associated with poikilocytes, especially acanthocytes, but low correlation coefficients suggested other factors were also involved. Cholesterol and phospholipid abnormalities in hepatic disease are another cause of acanthocyte or echinocyte formation [1], [2], [10], [13], [24]; we found no correlation with hepatic disease in this study, but the sample size was small. Whether the result of diet or disease, high plasma cholesterol causes cholesterol-enrichment of red cell membranes, expanding the outer leaflet of the phospholipid bilayer (forming membrane projections) and increasing membrane rigidity [18]. Decreased deformability and increased fragility shorten red cell lifespan and contribute to regenerative anemia [25].

An important finding in this study was the association of fragmentation with inflammation (often septic) and with malignant neoplasia in rabbits. This could be the result of bacterial toxins or microangiopathy, with endothelial fibrin deposition and microthrombi causing physical damage to erythrocytes [12], [14]. Fragmented erythrocytes usually occurred together with acanthocytes, which can undergo mechanical or “budding” fragmentation and which have been associated with fragmentation in dogs with hemangiosarcoma and glomerulonephritis [1], [12], [15]. Addition of Staphylococcal alpha toxin to suspensions of rabbit erythrocytes resulted in multiple, discrete surface blebs and finger-like protrusions (i.e., acanthocytes) that suggested separation of the cell membrane from the cell surface; human red cells were more resistant to this shape change [26]. In a murine model of bacterial sepsis, schistocytes (<1% of red cells) were observed 14 days post-infection and attributed to mild microangiopathic hemolysis [27].

Sepsis and inflammation-mediated oxidative damage and cytokines also alter red cell membranes, reducing deformability and increasing phagocytosis by macrophages [22], [27], [28]. Correlations between fragmentation and mild regenerative anemia and with heterophil, monocyte, fibrinogen, and globulins concentrations supported a relationship between bacterial infections, laboratory markers of inflammation, and red cell damage in the rabbits in our study. Lack of correlation of fragmented red cells with sample hemolysis was consistent with in vivo rather than in vitro fragment formation. Increased polychromatophils and fragments also contributed to high mean RDW and low MCH. High MPV and platelet counts supported reactive (inflammatory) thrombocytosis, in which IL-6-mediated thrombopoietin production stimulates platelet production [29]. False increases in MPV and platelet count from red cell fragments could have occurred in samples analyzed by the impedance analyzer in the first half of the study; however, the number of fragments was low.

Interestingly, high cholesterol diets can induce inflammation and oxidative stress together with acanthocyte formation in rabbits [30]. In one study [21] rabbits developed acanthocytes and fragments together with high concentrations of C-reactive protein, heterophils, and platelets, similar to our findings. The relationship between inflammation, hypercholesterolemia, and red cell morphology in rabbits fed nonatherogenic diets is unclear, but warrants further study.

We combined spherocytes, keratocytes, microcytes, and schistocytes as fragmented red cells because they often appeared together and in low numbers [12]. No samples contained a predominance or high % of spherocytes, as in immune-mediated hemolytic anemia. Keratocytes form after rupture of “blister cells” (seen rarely in our rabbits) or due to mechanical trauma; they are considered schistocytes by the International Council for Standardization in Hematology [20] and are frequently observed in dogs with concurrent echinocytosis or acanthocytosis [1].

Strong associations between echinocytes, renal disease, and electrolyte abnormalities suggested uremia as one mechanism of moderate to marked echinocytosis in rabbits, as in other species [14], [31]–[33]. Uremic toxins damage red cell membranes and lead to an influx of ionized calcium, externalization of phosphatidylserine in the lipid bilayer, and increased membrane rigidity concurrent with echinocyte formation [32]–[34]. In humans undergoing dialysis, % echinocytes correlated with intracellular calcium concentration and averaged 15–20% of RBCs [34]. The high anion gap in rabbits with echinocytosis and renal azotemia supported the presence of uremic acids. Increased echinocytes and red cell fragmentation in rabbits with renal disease also could have been associated with glomerulonephritis [1], [9], [12]. Increased plasma osmolality and hypernatremia (hypertonic dehydration) can also lead to echinocytosis, and may have been a factor in some rabbits in this study. Although smears in our laboratory are prepared soon after collection and dried rapidly, artifactual crenation likely contributed to echinocyte formation in some samples. Other disorders associated with echinocytosis in dogs (snakebite envenomation, lymphoma, cutaneous burns) [1], [9], horses (total body cation depletion) [1], [35], and rabbits (excess fluoride ingestion) [36] were not observed or associated with echinocytes in rabbits in this study.

A limitation of this retrospective study was the inability to verify underlying disease in all of the rabbits. In addition, we selected the primary disease process, but some rabbits had multiple problems. However, pathologic confirmation of disease was obtained in many of the rabbits, including diseases with potential relevance to poikilocyte formation. Further, many rabbits had diseases (such as fractures, dental malocclusion, and cystitis) that are readily diagnosed clinically or with imaging and laboratory tests. Despite this limitation, the large size of the database and consistent relationships between poikilocytes and clinicopathologic abnormalities helped support the primary findings of our study.

In conclusion, acanthocytes and echinocytes comprised >3% of erythrocytes in about one-third of healthy and diseased rabbits; a small proportion of rabbits had >30% poikilocytes. Echinocytosis also was associated with renal disease and electrolyte abnormalities, consistent with the effect of uremia on red cells. Rabbits with abscesses, other inflammatory disorders, and malignant neoplasia had more red cell fragmentation, which was associated with mild regenerative anemia and hematologic and biochemical evidence of inflammation. Serum cholesterol concentration also correlated poikilocytosis, but was not strongly predictive. Future research is warranted to prospectively evaluate the pathophysiologic mechanisms of poikilocyte formation, the role of diet, and the diagnostic and prognostic value of poikilocytes in rabbits.
